# Prediction of Uranium Adsorption Capacity in Radioactive Wastewater Treatment with Biochar

**DOI:** 10.3390/toxics12020118

**Published:** 2024-01-30

**Authors:** Zening Qu, Wei Wang, Yan He

**Affiliations:** College of Mechanical and Electrical Engineering, Northeast Forestry University, Harbin 150040, China; zeningqu@nefu.edu.cn (Z.Q.);

**Keywords:** wastewater treatment, uranium adsorption, biochar, prediction, meta-heuristic algorithms

## Abstract

Recently, Japan’s discharge of wastewater from the Fukushima nuclear disaster into the ocean has attracted widespread attention. To effectively address the challenge of separating uranium, the focus is on finding a healthy and environmentally friendly way to adsorb uranium using biochar. In this paper, a BP neural network is combined with each of the four meta-heuristic algorithms, namely Particle Swarm Optimization (PSO), Differential Evolution (DE), Cheetah Optimization (CO) and Fick’s Law Algorithm (FLA), to construct four prediction models for the uranium adsorption capacity in the treatment of radioactive wastewater with biochar: PSO-BP, DE-BP, CO-BP, FLA-BP. The coefficient of certainty (R^2^), error rate and CEC test set are used to judge the accuracy of the model based on the BP neural network. The results show that the Fick’s Law Algorithm (FLA) has a better search ability and convergence speed than the other algorithms. The importance of the input parameters is quantitatively assessed and ranked using XGBoost in order to analyze which parameters have a greater impact on the predictions of the model, which indicates that the parameters with the greatest impact are the initial concentration of uranium (C_0_, mg/L) and the mass percentage of total carbon (C, %). To sum up, four prediction models can be applied to study the adsorption of uranium by biochar materials during actual experiments, and the advantage of Fick’s Law Algorithm (FLA) is more obvious. The method of model prediction can significantly reduce the radiation risk caused by uranium to human health during the actual experiment and provide some reference for the efficient treatment of uranium wastewater by biochar.

## 1. Introduction

On 24 August 2023, Japan’s Fukushima Daiichi nuclear power plant initiated the discharge of nuclear-contaminated water into the sea. The wastewater, which originated from the operation and maintenance processes of the plant, contained more than 60 substances, such as radioactive uranium, tritium and plutonium. Uranium is the most essential fuel for nuclear reactors. Uranium mining, processing reactor operation and maintenance inevitably produce much radioactive uranium containing wastewater. Uranium is a substance with significant radiological, biological and chemical toxicity, and has been confirmed as a carcinogen. Its radioactive characteristics can destroy human DNA, meaning that the production of blood cells is affected and nerve cells are abnormal or dead, and then cause serious damage to the human hematopoietic organs, nervous system and reproductive system. Long-term exposure to uranium may increase the risk of malignant diseases such as lung cancer and blood cancer [1]. The World Health Organization (WHO) guidelines for drinking water quality specify a guideline value of 30 μg/L for uranium in drinking water [2], and the concentration of uranium in Fukushima nuclear wastewater can reach 5 mg/L, more than 160 times the World Health Organization’s guideline value for uranium in drinking water. The impact of radioactive wastewater on the ecosystem and human health is self-evident [3].

Therefore, it has become urgent to remove uranium from radioactive wastewater effectively. Various techniques such as adsorption [4], chemical precipitation [5], membrane separation [6] and ion exchange [7] have been used to treat uranium in aqueous solutions. Among all of these technologies, adsorption technologies tend to overcome several positive and negative aspects, especially when aligned with the waste material and the high-efficiency nature of the process. Among them, the adsorption approach is extensively utilized in the treatment of uranium-containing wastewater because it has the benefits of a low cost, easy operation and comprehensive source of materials. Mellah et al. [8] showed that activated carbon, a high-temperature biochar, is the oldest and most widely used adsorbent. In the last three years, researchers have been working on the invention of adsorbents for uranium. Cui et al. [9] synthesized a semiconductor covalent organic framework (NDA-TN-AO) characterized by outstanding photocatalytic and photovoltaic activity, enhancing uranium extraction by producing biotoxic reactive oxygen species. However, the preparation of this new material is both complex and expensive. In contrast, materials such as natural minerals and biochar are less costly and have a strong adsorption capacity.

Biochar is rich in carbon, and its surface pores and active functional groups can adsorb pollutants, playing a significant role in wastewater treatment [10]. The use of biochar for the efficient and practicable removal of uranium from radioactive wastewater has attracted a great deal of attention from scholars. The cost of biochar preparation is low, and its raw materials come from various sources, such as plant roots and stems, municipal and industrial organic wastes, etc. By converting these wastes into biochar, the effective utilization and recycling of resources can be realized, which has a positive significance for environmental protection. The different raw materials are composed of elements with varying ratios, resulting in differences in the physical and chemical features of biochar generated under the same experimental conditions, which can lead to different adsorption effects [11]. For example, wood biomass is high in carbon and hydrogen, while crop residues may be high in oxygen. Biochar generated from certain oxygen-rich biomasses may have a smaller specific surface area and less developed pore structure, resulting in weaker adsorption properties. Moreover, the adsorption effect of biochar is also affected by physical properties, chemical properties and experimental conditions. Parab et al. [12] adsorbed uranium in water with waste coconut shell fibers and studied the effect of parameters such as solution pH, adsorption time and temperature on the adsorption effect to find the optimum conditions for uranium adsorption. Guilhen et al. [13] investigated the impact of pyrolysis temperature on removing U(VI) from biochar. The results revealed that the carbon content grew with increasing pyrolysis temperature and the biochar produced at 350 °C reached 80% of the optimum removal efficiency of uranium from wastewater.

Improving the uranium adsorption capacity of biochar and its relationship with the performance parameters of biochar production are hot research topics nowadays. Traditional experiments on uranium adsorption in wastewater are complicated, time-consuming, and costly. However, researchers have accumulated a large amount of experimental data, enabling us to reveal these relationships through big data, artificial intelligence and algorithmic improvements, thereby decreasing the depletion of uranium and the radiation damage to the human body during the actual experiment. Generally speaking, many scholars use a single prediction model to predict adsorption capacity. This paper tries to predict the adsorption performance of biochar with multiple algorithmic models, and four algorithms with a BP neural network are selected to construct a model to predict the capacity of biochar to adsorb uranium.

Numerous scholars have chosen to use meta-heuristic algorithms (MHAs) in the study of adsorption capacity because this type of algorithm is a refinement of the heuristic algorithm and a product of integrating randomized algorithms with local search algorithms [14]. This type of algorithm is highly efficient and flexible, and is able to find the solution near the optimal answer in a shorter period, so in this paper, we also chose meta-heuristic algorithms. After checking many studies, it was found that the BP neural network is a frequently used artificial neural network model, which can adapt to different problems and data, and the neurons in it can be computed in parallel, so it has a better processing ability when dealing with large-scale data and tasks [15]. Compared with other methods, the combination of a meta-heuristic algorithm and the BP neural network can not only deal with complex problems better, but also further improve the reliability and practicability of the model. In this paper, we attempt to combine metaheuristic algorithms with the BP neural network, which can fully use their advantages, improve the performance of optimization algorithms, and be better applied to neural network training and optimization problems. Moreover, classical meta-heuristic algorithms are compared with new meta-heuristic algorithms, and so two classical algorithms with global search ability, namely Particle Swarm Optimization (PSO) and Differential Evolution (DE), and two new algorithms with global search ability:, namely Cheetah Optimization (CO) and Fick’s Law Algorithm (FLA), are selected. Among them, Particle Swarm Optimization (PSO) is simple and easy to implement, Differential Evolution (DE) has strong robustness, Cheetah Optimization (CO) has a wide range of applications, and Fick’s Law Algorithm (FLA) has strong interpretable ability. In this paper, the uranium adsorption capacities of the BP neural network and the different meta-heuristic algorithms are combined to forecast the uranium adsorption capacity of different biochar materials with numerous experimental data, and we quantitatively assess and rank the characteristic importance of the input parameters to provide some reference for the development of more efficient and environmentally friendly biochar treatments of uranium wastewater technology and products.

## 2. Materials and Methods

### 2.1. Data Collection and Preprocessing

In this paper, 777 sets of experimental data on uranium adsorption by biochar were collected from numerous studies [16,17,18,19,20,21,22,23,24,25,26,27,28], with 70% of the data being used as the training set and 30% of the data being used as the test set. Firstly, the data were preprocessed, including the identification and replacement of outliers, the inspection and supplementation of missing values, and data normalization to improve the quality of the dataset. The outliers were detected using the box-graph method, which distributed the data in boxes with different quantiles to show the distribution characteristics of the data. Outliers were considered as data points that fell outside or away from the box. Once an outlier was detected, we treated those outliers as missing values. To deal with outliers, we used the Newton interpolation method to construct a local interpolation polynomial, and replaced the outliers with the estimate of the polynomial at that point. Secondly, the data were normalized, and the min–max normalization method was adopted. Formula (1) transforms the original data linearly to map the result value to the specified range [29].

(1) $$X^{*}=\frac{X_{i}-X_{min}}{X_{max}-X_{min}}$$

In Formula (1), X_i_ represents the original data to be normalized, X* is the normalized value, X_max_ represents the maximum value of the initial data, and X_min_ is the minimum value. The normalization process scales the data so that the data are uniformly mapped to the [0, 1] interval and the original data distribution pattern can be maintained [30].

According to the experiments on uranium adsorption with biochar in many articles, the physical and chemical properties of biochar and the experimental conditions were taken as input parameters, and the uranium adsorption capacity of biochar was considered as the output parameter. The physical properties in the input parameters included the specific surface area (SA, m^2^/g), average pore size (Dav, nm), and total pore volume (VTot, cm^3^/g) of biochar. Chemical properties included the mass percentage of total carbon (C, %), the molar ratio of oxygen to carbon (O/C), and the molar ratio of oxygen to nitrogen ((O+N)/C). Experimental conditions included pH, temperature (T, K), initial concentration of uranium (C_0_, mg/L) and solid–liquid ratio (SLR, g/L) [31].

### 2.2. Meta-Heuristic Algorithms

#### 2.2.1. Particle Swarm Optimization Algorithm (PSO)

The concept of the Particle Swarm Optimization Algorithm originates from birds’ swarm foraging behavior. The solution of the optimization problem is abstracted into particles, and all the particles follow the current optimal particles, beginning from a series of initial solutions to seek the best solution, which is similar to the bird flocks constantly adjusting their direction to find food [32]. Assuming that the problem’s solution to be optimized is N-dimensional, i.e., the solution is related to all N elements, each particle has a position vector X_i_ = (X_i1_, X_i2_ … X_iN_) that changes as the number of iterations grows, corresponding to possible solutions to the question. At the same time, particle i also has a velocity vector V_i_ = (V_i1_, V_i2_ … V_iN_) of the same dimension as X that changes continuously as the iteration number becomes higher, which is used to determine which direction particle i moves from the current position and how far it moves, and the speed of all dimensions have an upper limit V_max_ [33]. The particle’s velocity as well as position update formulas are as in (2) and (3):

(2) $$V_{i}^{k+1}=wV_{i}^{k}+c_{1}r_{1}\left({pbest}_{i}^{k}-X_{i}^{k}\right)+c_{2}r_{2}\left({gbest}_{i}^{k}-X_{i}^{k}\right)$$

(3) $$X_{i}^{k+1}=X_{i}^{k}+V_{i}^{k+1}$$

where w denotes the inertia weight, which regulates the search of the solution space. c_1_ and c_2_ are learning factors that regulate the maximum step length of learning. r_1_ and r_2_ are random functions taking values in the scope of [0, 1] to increase the randomness. pbest represents the individual optimal position of the second particle, while gbest represents the optimal position search of the population. The inertia weight w indicates the effect of the previous speed vector on a new one. Referring to the parameter settings in many literatures, the parameter settings in this paper are as follows: inertia weight = 0.8, learning factor c_1_ = 0 for the self-knowledge part, and learning factor c_2_ = 0 for the social experience part. The maximum number of iterations is 100 and the algorithm terminates when it reaches the preset maximum number of iterations.

#### 2.2.2. Differential Evolutionary Algorithm (DE)

Differential Evolutionary Algorithm (DE) is an evolutionary algorithm similar to all other evolutionary algorithms; DE achieves the global search by means of a process of mutation, crossover and selection at each generation [34].

At the beginning of the optimization, a randomly initialized number of NP D-dimensional parameter vectors X is used, while X_i_ denotes the ith solution, X_i_ = (X_i,1_, X_i,2_ … X_i,D_). After initialization, the individuals of each parent obtain their offspring through a mutation strategy, and for each solution vector X_i_, the corresponding mutation vector V is denoted as Equation (4):

(4) $$V_{i}=X_{r_{0}}+F\left(X_{r_{1}}-X_{r_{2}}\right)$$

In this formula, r_0_, r_1_ and r_2_ are three dissimilar stochastic numbers belonging to [1, …, NP]. F is the variation operator, which takes the value in the range of [0, 2]. If F is too small, it might be trapped in a local optimum, while if F is too big, it is not prone to convergence. After the mutation is completed, the crossover operation is performed on the generated individuals as follows (5):

(5) $$u_{i,j}=\left\{\begin{array}{c} v_{i,j},\mathrm{if}\;\left(rand\leq Cr\right)\\ x_{i,j},\mathrm{if}\;\left(rand>Cr\right) \end{array}\right.$$

In this formula, Cr is the crossover operator, a random number within [0, 1], which is used to control the selection of the variant vector value or the original vector value. After the crossover operation, according to the fitness of the individual, the better individual is selected to enter the next generation, so that the better adapted offspring or parent individuals form a new population and continue to loop iterations until the stopping requirement is met. Referring to the parameter settings in many studies, the parameters are established as follows in this paper: variation operator F = 0.5, crossover operator Cr = 0.7. The maximum number of iterations is 100, and when the algorithm reaches the maximum number of iterations, the algorithm is terminated.

#### 2.2.3. Optimization Algorithm (CO)

The concept of the Cheetah Optimization Algorithm comes from the hunting behavior of cheetahs, which can detect prey when they patrols or scan their surroundings. Upon seeing its prey, the cheetah may stay in its original position and begin to attack when the prey approaches it. In short, it is divided into three strategies: searching, sitting and waiting, and attacking [35]. During hunting, the search or attack strategy is deployed randomly. Assume that the search probability is r_1_, the vector update probability is r_2_, and the position update probability is r_3_; r_1_, r_2_ and r_3_ are homogeneous arbitrary numbers from [0, 1]. If r_2_ ≥ r_3_, the cheetah chooses the motionless waiting strategy; otherwise, it chooses the find or offensive strategy.

The cheetah searches according to the location of the prey and the surrounding environment, assuming that the problem’s solution to be optimized is D-dimensional and the number of cheetahs is n. The location of the cheetah is based on Equation (6), which has been updated.

(6) $$X_{i,j}^{t+1}=X_{i,j}^{t}+{\hat{r}}_{i,j}^{-1}\cdot \alpha_{i,j}^{t}$$

In this equation, t indicates the hunting time, $X_{i,j}^{t}$ denotes the current location of cheetah i in alignment j(j = 1, 2, …, D), ${\hat{r}}_{i,j}^{-1}$ denotes the randomized parameter of cheetah i listed as j, and $\alpha_{i,j}^{t}$ denotes the step size of cheetah i with arrangement j. In order to prevent the prey from noticing its presence, the cheetah then adopts a sit-and-wait strategy, i.e., it keeps its position unchanged and waits until the prey approaches. When the time is right for hunting, the cheetah will adjust its moving direction in time according to the position of the prey and then capture the prey; the cheetah’s position in the attack updates the formula as follows (7):

(7) $$X_{i,j}^{t+1}=X_{B,j}^{t}+{\overset{ˇ}{r}}_{i,j}\cdot \beta_{i,j}^{t}$$

In this equation, t indicates the hunting time, $X_{B,j}^{t}$ denotes the current location of cheetah i in arrangement j (j = 1, 2, …, D) in the current position, ${\overset{ˇ}{r}}_{i,j}$ denotes the steering related to cheetah i in alignment j, and $\beta_{i,j}^{t}$ denotes the correlation interaction factor with cheetah i in alignment j. Referring to the parameter settings in many studies, the parameter settings in this paper are r_1_ = 0.8, r_2_ = 0.5, r_3_ = 0.9. The maximum number of iterations is 100. When the algorithm reaches the preset maximum number of iterations, the algorithm terminates.

#### 2.2.4. Fick’s Law Algorithm

Fick’s Law Algorithm is a meta-heuristic algorithm motivated by Fick’s Law. Fick’s law is a fundamental law describing the diffusion process of substances, i.e., molecules tend to spread from areas of high density to areas of low density. FLA takes advantage of the diffusion property to optimize the search. The fundamental idea of the algorithm is to consider the problem space as a finite-dimensional space in which the optimal solution is searched by modeling the process of substance diffusion [36].

Assuming that the problem’s solution to be optimized is D-dimensional and the number of solutions is N, the randomly generated initial population is first divided into two equal groups. According to Fick’s Law, the diffusion operator updates the individuals’ position, including transferring between three phases: the detective phase, the transition phase from exploration to exploitation, and the exploitation phase. The updated fitness value is obtained by calculating from the new position. Then, the global optimal solution is updated according to the fitness value until the iteration is stopped when the termination condition is reached. The maximum number of iterations is 100 and the algorithm terminates when it reaches the preset maximum number of iterations.

### 2.3. BP Neural Network

The BP neural network, also called back propagation neural network, is a typical neural network training algorithm. The BP neural network consists of forward propagation and back propagation, in which forward propagation is when the input layer outputs the result and error after inputting samples through the transfer function of the hidden layer and the output layer. Back propagation is when the error is assigned to each neuron, and then each neuron adjusts the weights and thresholds so that the input and output error reach the target value [37]. The BP neural network model is highly error-tolerant, self-learning and self-adapting, which overcomes the difficulties of model building and parameter estimation in traditional prediction methods.

**Figure 1:**
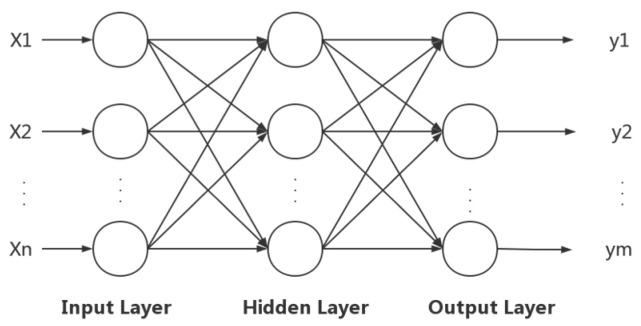
Schematic diagram of BP neural network propagation.

The BP neural network mainly consists of three layers, including the input layer, the hidden layer, and the output layer of the BP neural network. The propagation principle is shown in Figure 1. In Figure 1, X_1_, X_2_, …, X_n_ are the input samples of the BP neural network and y_1_, y_2_, …, y_m_ are the output variables of the BP neural network [38].

### 2.4. Model Construction

Four prediction models are constructed in Python 3.10: PSO-BP, DE-BP, CO-BP and FLA-BP. Firstly, the construction of the BP neural network needs to define the structure of the network, including the number of neurons in the input layer, the hidden layer and the output layer, the number of iterations and other parameters, and establish the initial weight and bias. The BP neural network uses a 3-layer hidden layer structure to capture the important features of the input data without over-complexity. It has an input dimension of 10, an output dimension of 1, a learning rate of 0.01, and a number of iterations of 1000. Forward propagation is carried out according to the training data, the output value is calculated, and then the error is calculated according to the output value and the label of the training data. Based on the error value, the error is backpropagated from the output layer, and the weights and biases are updated continuously until the network reaches an acceptable error range or the training reaches a predetermined number of rounds. Second, in order to make the BP neural network converge faster and achieve higher accuracy, this paper used the Particle Swarm Optimization algorithm (PSO), the Differential Evolution algorithm (DE), the Cheetah Optimization algorithm (CO) and Fick’s Law Algorithm (FLA) to optimize the BP neural network, respectively, to replace the original gradient descent method, and constructed four prediction models, such as PSO-BP. According to the search results of the four algorithms, the weights and biases of the BP neural network were updated, the parameters of the BP neural network were adjusted, and several iterations were performed to improve the model’s performance.

### 2.5. Performance Assessment Measures

In this paper, the accuracy of the BP neural network-based model was determined by the coefficient of determination (R^2^) and the error rate. The error rate includes the mean square error (MSE), mean absolute error (MAE) and mean bias error (MBE) [39]. The specific formulas are shown in (8)–(11):

(8) $$R^{2}=\frac{SSR}{SST}=1-\frac{SSE}{SST}=1-\frac{\sum_{i=1}^{N}{\left(y_{i}-\hat{y_{1}}\right)}^{2}}{\sum_{i=1}^{N}{\left(y_{i}-\bar{y}\right)}^{2}}$$

(9) $$MSE=\frac{1}{N}\sum_{i=1}^{N}{\left(y_{i}-\hat{y_{1}}\right)}^{2}$$

(10) $$MAE=\frac{1}{N}\sum_{i=1}^{N}\left|y_{i}-\hat{y_{1}}\right|$$

(11) $$MBE=\frac{1}{N}\sum_{i=1}^{N}\left(y_{i}-\hat{y_{1}}\right)$$

In Equations (8)–(11), SSE is the sum of squares of residuals, SST is the total sum of squares and the actual value, y_1_, y_2_, …, y_n_ are the true values, $\bar{y}$ is the average of all the true values, ${\hat{y}}_{1},{\;\hat{y}}_{2}\ldots {\hat{y}}_{n}$ are the predicted values, and $y_{i}-\hat{y_{1}}$ are the residuals of the ith sample, which indicate the difference between predicted and valid values, and provide a greater reflection of the reality of the error in the predicted value.

## 3. Results and Discussion

### 3.1. Model Prediction Results

**Figure 2:**
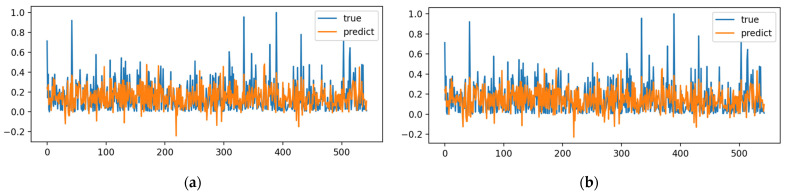

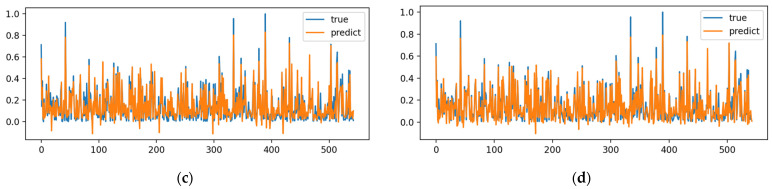
Training set fitting results of biochar for predicting the uranium adsorption capacity: (**a**) PSO-BP; (**b**) DE-BP; (**c**) CO-BP; (**d**) FLA-BP.

Figure 2 shows the fitting results of the training set data after the training of the BP neural network using four optimization algorithms. The number of iterations is 1000 and the learning rate is 0.01.

As illustrated in Figure 2, the particle swarm optimization algorithm and differential evolution algorithm cannot fully fit data with significant mutation and variance. The main reason for this is that the Particle Swarm Optimization and Differential Evolution Algorithms tend to fall into local optimal solutions when facing high-dimensional optimization problems, which also leads to instability of the optimization results. The Cheetah Optimization Algorithm and Fick’s Law Optimization Algorithm proposed in the past two years show strong performance in jumping out of the local optimal solution. As can be seen from Figure 2, both algorithms exhibit extremely high fitting ability in the face of extreme data, mainly due to their intelligent search strategy, their ability to avoid falling into local optimality, their ability to handle extreme data, and their adaptive parameter adjustment mechanism. The convex optimization ability of the Fick’s Law Optimization Algorithm can reach the same level as that of the Adam optimizer, showing obvious superiority.

**Figure 3:**
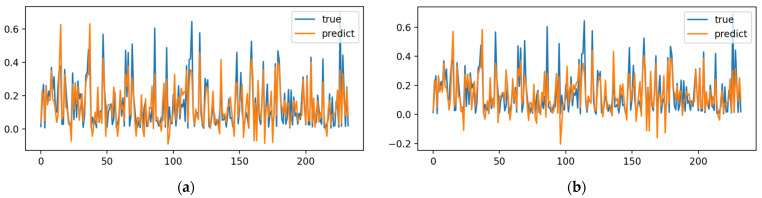

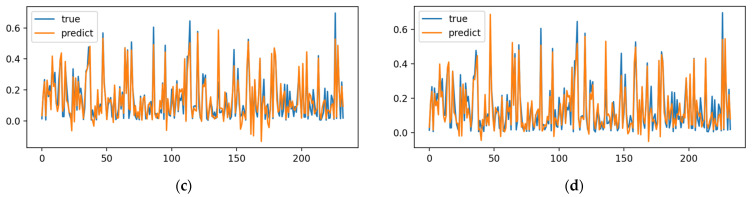
Test set fitting results for predicting uranium adsorption capacity of biochar: (**a**) PSO-BP; (**b**) DE-BP; (**c**) CO-BP; (**d**) FLA-BP.

Figure 3 shows the schematic diagram of fitting results of the trained BP neural network to the test set. As opposed to the training set, the Fick’s Law Optimization Algorithm has more obvious advantages, showing strong generalization performance and adaptability to various complex scenarios. After analyzing the experimental results, it is concluded that the Fick’s Law Algorithm is stronger than the other three algorithms in terms of its robustness, generalization and capacity to go beyond the local optimum, and can achieve the optimization performance of the mainstream optimizer and can be used as an alternative to the mainstream optimizer. Its randomness can provide more feasible solutions for BP neural networks. At present, the only shortcoming is that the Fick’s Law Optimization Algorithm has a shorter optimization time compared with mainstream optimizers. In the future, studies will concentrate on solving the problem of insufficient convergence speed. The leading solution can be to improve its initialization method to have a lower initial fitness value, accelerating its convergence speed.

### 3.2. Comparative Analysis of Model Performance

**Figure 4:**
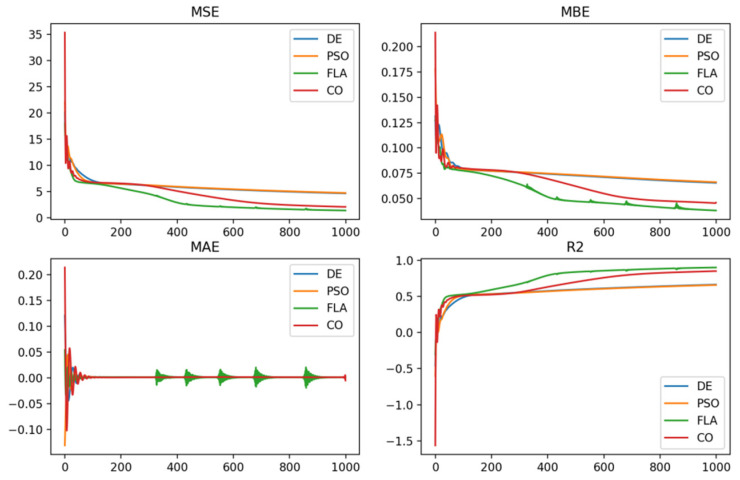
Training process index diagram.

The optimization behavior of the algorithm is quantitatively analyzed by analyzing the evaluation indicators. The changes in each indicator in the training process are shown in Figure 4. The mean square error (MSE) represents the mean of the square of the difference between the original and predicted values in the dataset to measure the variance of the residual. The mean absolute error (MAE) represents the mean of the absolute difference between the actual and predicted values in the dataset, as measured by the mean of the residual in the dataset. The mean bias error (MBE) allows one to know the size of the deviation between the predicted result and the actual value, with positive deviation indicating that the data error is overestimated and negative deviation indicating that the error is underestimated.

From the MSE index in Figure 4, we can establish that the minimum value of the Fick’s Law Optimization Algorithm (FLA) is 0.00424, indicating the best fitting effect, followed by the Cheetah Optimization Algorithm (CO), whose value is 0.00555. Since MSE is used as a loss function to perform optimization of the BP neural network in this experiment, all indexes except for MSE exhibit the characteristics of oscillating convergence. As can be seen from Figure 4, the Fick’s Law Optimization Algorithm (FLA) and the Cheetah Optimization Algorithm (CO) have better convergence accuracy in terms of the MAE and MBE indexes. The Fick’s Law Optimization Algorithm (FLA) is the best, followed by the Cheetah Optimization Algorithm (CO).

R^2^ explains the variance score of the regression model. According to Table 1, the R^2^ after optimization of the Fick’s Law Optimization Algorithm (FLA) is 0.90. The R^2^ after the Cheetah Optimization Algorithm (CO) is 0.85. An R^2^ closer to 1 indicates that the independent variable explains the variance change in the dependent variable better. It can be concluded from Figure 4 that the models of the Fick’s Law Optimization Algorithm (FLA) and the Cheetah Optimization Algorithm (CO) outperform the Particle Swarm Optimization Algorithm (PSO) model and the Differential Evolutionary Algorithm (DE) model. But when predictive models are in pursuit of higher accuracy, they often encounter some tradeoffs or limitations. For example, overfitting can reduce a model’s ability to generalize because it makes the model too dependent on specific details in the training data.

### 3.3. Performance Analysis in CEC Tests

The test results of CEC2005 are widely recognized and can be used as a standard to assess the performance of optimization algorithms with high levels of authority. The CEC2005 test set contains 25 test problems, i.e., $f_{1}\left(x\right)-f_{25}\left(x\right)$, contains single-peak test functions $f_{1}\left(x\right)-f_{5}\left(x\right)$, basic multi-peak test functions $f_{6}\left(x\right)-f_{12}\left(x\right)$, extended multi-peak test functions $f_{13}\left(x\right)-f_{14}\left(x\right)$, and hybrid composite test functions $f_{6}\left(x\right)-f_{12}\left(x\right)$ [40]. The CEC2005 test can provide some reference value, but it cannot completely replace the need for performance evaluation in real applications. On the one hand, the set of functions in the CEC2005 test is designed for evaluating the performance of optimization algorithms, not for simulating problems in real applications. On the other hand, the constraints, boundary conditions and objective functions of real problems may be more complex and may involve more decision variables and influencing factors.

**Figure 5:**
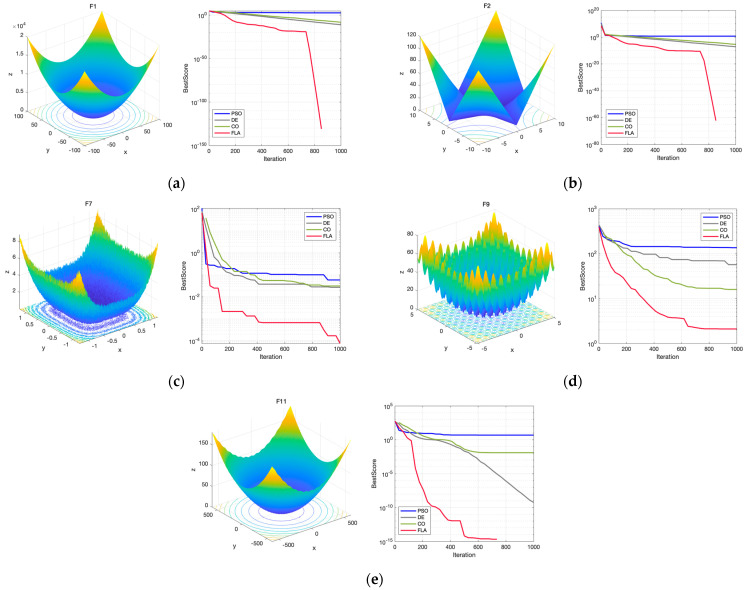
CEC test results: (**a**) F1; (**b**) F2; (**c**) F3; (**d**) F4; (**e**) F5.

The single-peak test functions, $f_{1}\left(x\right)-f_{5}\left(x\right)$, are utilized to examine further the algorithm’s ability to develop on the single-peak test function and the convergence precision of the algorithm. Unlike the single-peak test function, the multi-peak test function has more than one local optimal solution, and the function values corresponding to each local optimal solution may differ significantly. The prediction model constructed in this study is assigned 10 input parameters, so the test functions $f_{1}\left(x\right)-f_{14}\left(x\right)$ applicable to low-dimensional optimization problems are selected for analysis, and some of the analysis results are as follows:

As shown in Figure 5, the convergence speed of the Fick’s Law Algorithm (FLA) is substantially and significantly better than that of the Particle Swarm Optimization Algorithm (PSO), Differential Evolutionary Algorithm (DE) and Cheetah Optimization Algorithm (CO). Moreover, from the trend of curve fitting, FLA has a stronger local development ability than other models and a better search ability than other algorithms when dealing with high-dimensional complex problems. Through comprehensive comparative analysis and verification, it is concluded that the FLA solution’s accuracy and stability are high, it has a faster convergence rate, and its advantages are significant.

### 3.4. Important Feature Visualization

**Figure 6:**
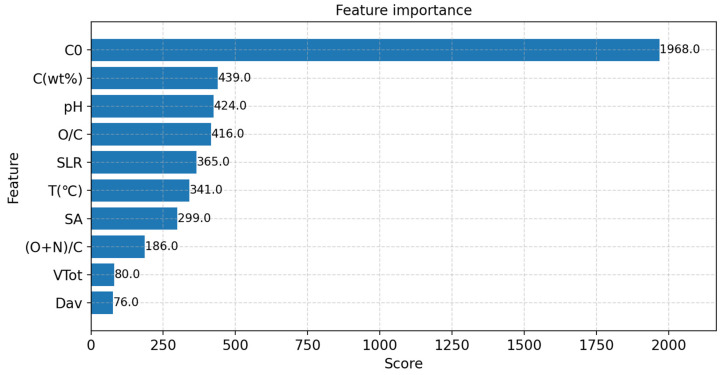
BP feature importance score plot.

Feature importance assessment quantifies feature importance during training by documenting the total number of splits and the average message yield of the characteristics. This critical task in machine learning helps to analyze which input parameters significantly impact the model’s prediction results [41]. This study uses feature importance assessment to investigate which features have a greater impact on the model’s predictive performance, thereby optimizing data collection and analysis strategies. In this study, the input feature X and the target variable Y are subjected to data normalization, i.e., the data are transformed into a regular normal distribution with mean 0 and variance 1. Then, the importance scores of the features are calculated using XGBoost for the trained model, and the score range is chosen from 0 to 2000 to show the degree of importance clearly. Finally, the feature scores are plotted from the most significant to the most minor using Matplotlib, as shown in Figure 6.

Among the experimental conditions, the most influential on the ability of biochar to adsorb uranium is the initial concentration of uranium (C_0_, mg/L), which has a value of 1968, and the adsorption rises with the preliminary concentration of uranium. It begins to slow down when the initial concentration of uranium is too large. The second most influential is pH, with a value of 424. pH indicates the acidity or alkalinity of the solution in the environment. Under different pH conditions, the charge state of the biochar surface changes. When the pH is low (acidic environment), the surface of biochar is positively charged and attracted to the negative charge of uranium. In contrast, at a higher pH (alkaline environment), the surface of the biochar is negatively charged and repelled by the negative charge of uranium, resulting in lower adsorption capacity. In practical applications, selecting appropriate pH conditions can enhance the adsorption effect of biochar on uranium.

The most influential chemical property is the mass percentage of total carbon (C, %), which has a value of 439, and the mass percentage of carbon in biochar indicates the degree of carbonization of the biomass. A higher mass percentage of carbon in biochar indicates a higher number of adsorbable groups, thus increasing the adsorption capacity. Next is the molar ratio of oxygen to carbon (O/C), which has a value of 416. When the molar ratio of oxygen to carbon is high, it indicates that the oxygen content in the biochar is relatively high, which may compete with uranium adsorption and reduce the adsorption effect. Therefore, lower molar ratios of oxygen to carbon and higher mass percentages of carbon are usually favorable for increasing the adsorption capacity of biochar for uranium.

The most influential of the physical properties is the specific surface area (SA, m^2^/g) of the biochar, with a value of 299, whose magnitude visually indicates the magnitude of the adsorption capacity of the adsorbent, and a greater specific surface area implies more reactive adsorption sites, which can increase the contact area between the biochar and the uranium, and thus enhance the adsorption effect. Next is the total pore volume (VTot), which has a value of 80. A larger total pore volume means more adsorption space, providing more sites to accommodate uranium ions. This makes the biochar more effective in adsorbing uranium. Therefore, a larger specific surface area and total pore volume can improve the adsorption ability of biochar for uranium when manufacturing adsorbents. These properties make biochar an effective adsorbent material with many applications in water treatment and environmental remediation.

## 4. Conclusions

In this paper, four meta-heuristic optimization algorithm models based on the BP neural network are constructed to predict the uranium adsorption capacity of biochar, providing some lessons for the efficient management of uranium wastewater.

1. A prediction model of the uranium adsorption capacity is constructed using Python 3.10. Four meta-heuristic optimization algorithms for model searching based on the BP neural network, namely Particle Swarm Optimization (PSO), Differential Evolution (DE), Cheetah Optimization (CO) and Fick’s Law Algorithm (FLA), are used to establish four prediction models: PSO-BP, DE-BP, CO-BP and FLA-BP. Predictive models are available to foresee the uranium adsorption capacity of biochar in actual situations, significantly reducing the experimental effort and safety risks associated with radioactivity;
2. The accuracy of the four models is verified by the coefficient of determination (R^2^) and error rate. After training and validation, the Fick’s Law Algorithm (FLA) is optimized with R^2^ of 0.90, showing more obvious advantages and a strong generalization performance. This algorithm is more robust than the other three regarding its robustness, generalization, and ability to go beyond the local optimum. When predictive models are in pursuit of higher accuracy, there are often trade-offs or limitations that require continued research;
3. The influence of the input performance parameters in the prediction model on the adsorption capacity is analyzed using XGBoost to search for the optimal performance parameters. The analysis showed that the most influential experimental conditions on the ability of biochar to adsorb uranium are the initial concentration of uranium (C_0_, mg/L) and pH; the most influential chemical properties are the mass percentage of total carbon (C, %) and the molar ratio of oxygen to carbon (O/C); and the most influential physical properties are the specific surface area of biochar (SA, m^2^/g) and the total pore volume (VTot), providing some important lessons for the study of the uranium adsorption capacity of biochar.

## Figures and Tables

**Table 1:** Training process index comparison table.

	MSE	MBE	MAE	R^2^
PSO-BP	0.01106	0.00097	0.07655	0.59230
DE-BP	0.01348	0.00172	0.08292	0.50359
CO-BP	0.00555	0.00098	0.05355	0.79556
FLA-BP	0.00424	0.00082	0.04816	0.90125

## Data Availability

Data are available upon request from the corresponding author.
